# A Genome-Wide Association Study of a Korean Population Identifies Genetic Susceptibility to Hypertension Based on Sex-Specific Differences

**DOI:** 10.3390/genes12111804

**Published:** 2021-11-16

**Authors:** Seong-Beom Cho, Jinhwa Jang

**Affiliations:** 1 Department of Biomedical Informatics, College of Medicine, Gachon University, Incheon 21565, Korea; 2 Department of Biomedical Informatics, Center for Genome Science, National Institute of Health, KCDC, Osong, Cheongju-si 28159, Korea; jinhjang@korea.kr

**Keywords:** hypertension, sex, genome-wide association study, genetic effect, heterogeneity

## Abstract

Genome-wide association studies have expanded our understanding of the genetic variation of hypertension. Hypertension and blood pressure are influenced by sex-specific differences; therefore, genetic variants may have sex-specific effects on phenotype. To identify the genetic factors influencing the sex-specific differences concerning hypertension, we conducted a heterogeneity analysis of a genome-wide association study (GWAS) on 13,926 samples from a Korean population. Using the Illumina exome chip data of the population, we performed GWASs of the male and female population independently and applied a statistical test that identified heterogeneous effects of the variants between the two groups. To gain information about the biological implication of the genetic heterogeneity, we used gene set enrichment analysis with GWAS catalog and pathway gene sets. The heterogeneity analysis revealed that the rs11066015 of ACAD10 was a significant locus that had sex-specific genetic effects on the development of hypertension. The rs2074356 of HECTD4 also showed significant genetic heterogeneity in systolic blood pressure. The enrichment analysis showed significant results that are consistent with the pathophysiology of hypertension. These results indicate a sex-specific genetic susceptibility to hypertension that should be considered in future genetic studies of hypertension.

## 1. Introduction

Hypertension is a major global health challenge because of its high prevalence and complications including cardiovascular disease and premature death [1,2,3]. The development of hypertension has genetic susceptibility [4,5,6]. Recently, genome-wide association studies (GWASs), in which hundreds of thousands of genetic variants are genotyped and analyzed for disease association, have revealed genes associated with hypertension [7]. Over five thousand single nucleotide polymorphisms (SNPs) that are significantly associated with hypertension have been reported in the NHGRI-EBI GWAS catalog to date [8]. Hypertension is also influenced by other factors such as diet, alcohol intake, smoking, obesity, stress, age, and sex [9] and it is important to understand the interaction between genes and such factors.

Among these factors, sex is known to affect blood pressure and hypertension [9,10]. Sex hormones, including estrogen and androgen, are a primary factor for the sex difference in the development of hypertension because they affect vascular tone, smooth muscle cell growth, and the sympathetic nervous system. These effects are mediated by a sex hormone receptor-mediated signaling pathway in endothelial and vascular smooth muscle cells [11]. Although previous research has reported controversial findings, the increased incidence of hypertension in the post-menopausal period supports the role of estrogen in the development of hypertension [11].

During the past decade, GWASs have revealed many genetic susceptibility loci for chronic diseases. Moreover, they have identified sex differences in the genetic susceptibility of chronic diseases. For example, Hartiala et al. found that rs715 of carbamoyl-phosphate synthase 1 is associated with decreased risk of coronary artery disease only in women [12]. Such sex differences have been observed in the sex-stratified GWASs of type 1 and type 2 diabetes [13] and Crohn’s disease [14]. In the GWAS of rheumatoid arthritis, some loci showed strong associations in both sexes, while others showed different significances between male and female groups [15]. Several studies have reported both common and rare variants with a significant genome-wide association in hypertension using an exome array or sequencing platform [16,17,18,19,20]. However, no GWASs have ever identified genetic variants that showed a sex-specific effect on hypertension or blood pressure. The aim of this study was to identify genetic loci that have differential susceptibility to hypertension and blood pressure based on sex. We conducted exome-wide association analyses for hypertension, systolic blood pressure (SBP), and diastolic blood pressure (DBP) in a Korean population of 13,926 individuals. We performed GWASs of the three phenotypes using the whole population. We also performed sex-stratified GWASs and tested the heterogeneity of genetic effects on the phenotypes between the sexes. Then, gene set enrichment analysis using GWAS catalog gene sets, and pathway information was applied to identify functional implications for the GWAS results.

## 2. Materials and Methods

To develop an infrastructure for preventive health care using genetic information, the National Institute of Health, KCDC, initiated a prospective cohort study, named the Korean Genome and Epidemiology Study (KoGES) project [21]. The project was designed to collect participants predominantly aged from 40–69, although a younger population was included. Blood pressure (BP) was measured using a standard mercury sphygmomanometer after participants had been in a sitting position for at least 15 min. Hypertensive status was defined as SBP ≥ 140 mmHg or diastolic DBP ≥ 90 mmHg or whether hypertension had been treated with anti-hypertensive medication. All participants provided written informed consent, and the study was approved by the Institutional Review Board of the National Institute of Health, KCDC.

We used the Illumina Exome BeadChip (San Diego, CA, USA) array data of 13,926 study participants from the three population-based cohorts of the KoGES project. We used 7524 participants from the Ansung and Ansan (AS) cohort, 3434 participants from the Health Examinee (HEXA) cohort, and 3068 participants from the Cardiovascular Disease Association Study (CAVAS) cohort which had exome array genotype data. Participants having missing values were filtered (n = 100). The information for each cohort is described in reference [21]. The Illumina Exome BeadChip is a genotyping array platform that includes variants represented by 241,901 SNPs selected from the human exome region [22]. This chip is focused on protein-altering variants (non-synonymous, stop and splice variants) and includes other variants such as GWAS hotspots of previous studies, mitochondrial SNPs, and ancestry informative markers. The exome array data of the current analysis was produced in previous research identifying pleiotropic loci for cardiometabolic traits [23]. We used the filtered exome array dataset based on the quality control of the original research. In this analysis, all genomic information is based on the Genome Reference Consortium Human Build 37.

Association analysis for hypertension and BP was performed under an additive genetic model. The genetic effect of each exonic variant was adjusted for age, sex, and body mass index (BMI) in the total population. In the sex-stratified analysis, age and BMI were used as covariates. We applied the linear regression model for the identification of genetic variants that affect SBP and DBP. We also used the logistic regression model to find genetic loci that are susceptible to hypertension. In the quantitative trait loci analysis of BP, we excluded participants who were taking anti-hypertensive medication to rule out any potential bias resulting from such medication. Genomic-control corrected *p*-values were used to determine statistical significance. All of the association analysis and clumping of variants after the association analysis were performed according to the default parameters in the PLINK program [24].

For the identification of the heterogeneity of genetic loci, we performed sex-stratified GWASs for hypertension in the male and female groups independently. We used the Cochran *Q* statistic to identify the heterogeneity of genetic effects between males and females. The Cochran *Q* statistic has been widely used for the identification of genetic heterogeneity in the meta-analysis of GWASs [25]. It detects the heterogeneity of genetic effects of a variant from different studies, which is caused by ancestries, linkage disequilibrium patterns, sub-phenotypes, ages of disease onset, family history of disease, or gender. In this analysis, we considered significant Cochran’s *Q* statistics as an indicator for heterogeneity of genetic effects of variants between males and females in the development of hypertension. The Cochran’s *Q* statistic was computed using the getmstatistic R package [26], and statistical testing was performed using Chi-square distribution. The degree of freedom was set to one because the heterogeneity was assessed between two genetic loci from the male and female groups. Multiple testing correction was performed with Bonferroni’s method. In the heterogeneity test, we selected loci such that the results of the association study were nominally significant in at least one of the sex groups.

To identify the biological implications of the heterogeneity analysis results, we applied the functional annotation of SNPs. For this purpose, the FUMA web tool was used [26]. We used the ‘SNP2GENE’ function for the application of Multi-marker Analysis of GenoMic Annotation (MAGMA) gene set analysis [27]. The genomic-control corrected *p* values were used as input data. We also used the ‘GENE2Function’ of the FUMA web tool for the functional annotation.

## 3. Results

### 3.1. Significant Results of the Exome-Wide Association Study in the Total Population

Although the main purpose of this study was to reveal differences in genetic susceptibility to hypertension based on sex, we first carried out an exome-wide association study (EWAS) for hypertension and BP in the total population. The total sample size for the analysis was 13,926 (male = 6402, female = 7524). Before the EWAS, we imputed the exome data using Beagle with default parameters [28]. The imputation was performed only for the loci that had less than 10% of missing genotypes in the total population. In total, 50,543 variants were used in the analysis.

In the EWAS of hypertension in the total population, three variants (rs16998073, rs12413409, and rs2681472) showed exome-wide significance (Bonferroni’s adjusted *p*-value < 9.89 × 10^−7^, Table 1). These variants had been identified in previous GWASs for BP and hypertension. For example, the rs16998073 that is located at the intergenic region of PRDM8, FGF5, showed genome-wide significance for SBP and DBP [29,30]. The significance was independently reproducible in the genetic association analysis of hypertension with a Chinese population [31]. The other two variants also showed a significant result in previous GWASs [32,33]. In the total population EWASs for SBP and DBP, there were no exome-wide significant results with the adjusted *p* value.

The MAGMA analysis of hypertension in the total population identified four susceptibility genes for hypertension with Bonferroni’s multiple testing correction (adjusted *p*-value threshold = 0.05/10,536 = 4.75 × 10^−6^, Table 2). In the result, the CNNM2 gene showed the strongest significance (*p* = 1.5 × 10^−8^). This is consistent with the single SNP analysis because rs12413409 of CNNM2 was significant in the single SNP-wise EWAS of hypertension (Table 1).

### 3.2. Results of Sex-Stratified EWASs

**Figure 1:**
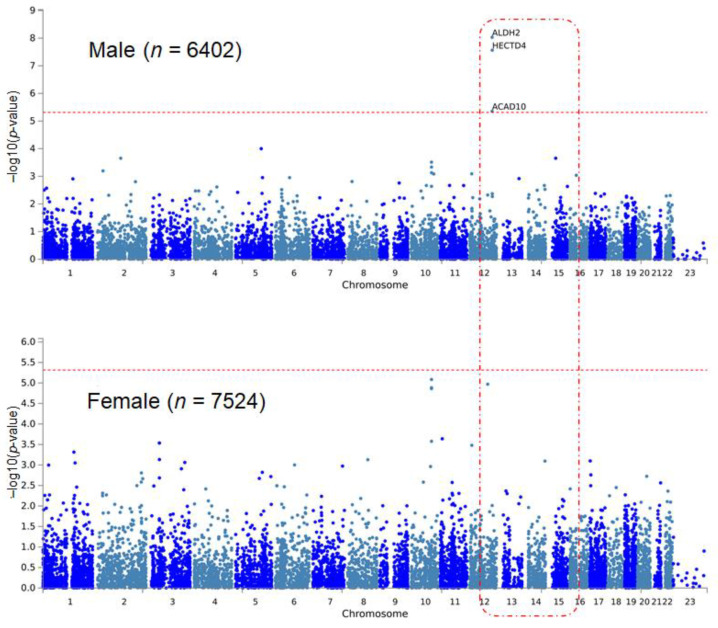
Manhattan plots of sex-stratified MAGMA analysis for hypertension. The Y-axis indicates −log_10_ (*p*-values), where *p*-values come from MAGMA analysis. Note that ALDH2, HECTD4, and ACAD10 are significant only in the male population. The red horizontal dotted line indicates the cutoff threshold for statistical significance.

In the sex-stratified EWAS for hypertension, four variants were significant (Table 1). Three of them were significant only in males, and one variant showed significance only in females. Among them, the rs11066015 of acyl-CoA dehydrogenase family member 10 (ACAD10) gene showed the greatest significance in the sex-stratified EWAS (odds ratio (OR) = 0.73, *p* = 7.99 × 10^−9^), and the result was significant only in the male group. This variant was identified as a genome-wide significant locus for coronary artery disease and esophageal cancer [34,35]. The rs16998073, which was the most significant variant for hypertension in the total population and significant only in the female population, showed the second greatest significance (OR = 1.25, *p* = 1.18 × 10^−7^). The significance of rs16998073 was not reproducible in males. The rs11066280 of HECT domain E3 ubiquitin protein ligase 4 (HECTD4) gene showed exome-wide significance in males only (OR = 0.75, *p* = 1.26 × 10^−7^). It has been reported that this variant is associated with both SBP and DBP in previous GWASs [36,37]. The rs1392550 was a novel variant for hypertension, significant only in the male group (OR = 1.22, *p* = 8.77 × 10^−7^). MAGMA analysis identified significant genes for hypertension in males (Table 2, Figure 1). In this group, three genes (ALDH2, HECTD4, and ACAD10) were significant with Bonferroni’s multiple testing correction (adjusted *p*-value < 3.84 × 10^−6^, Table 2). There was no significant MAGMA result in the female population.

**Figure 2:**
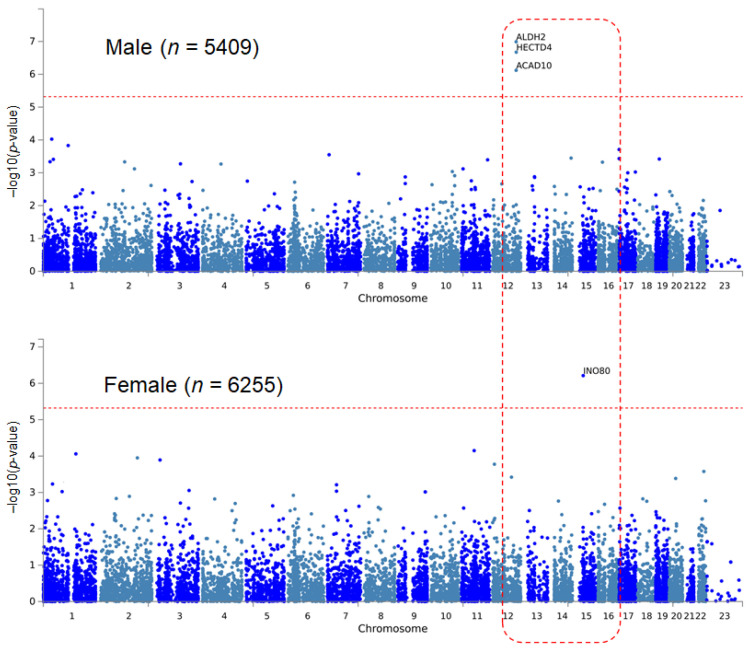
Manhattan plots of sex-stratified MAGMA analysis for systolic blood pressure. As in the same analysis for hypertension, ALDH2, HECTD4, and ACAD10 show significant results only in the male population.

In the sex-stratified EWAS of the male group for SBP, variants that were significant in hypertension in males (rs11066015, rs11066280) showed significant results (Table 3). In females, rs142469845 of INO80 was significant. This SNP is a rare variant whose minor allele frequency was < 0.01; therefore, the effect size of the variant was far greater than that of the other variants (β = 42.93, *p* = 6.15 × 10^−^^7^). MAGMA revealed four significant results in the sex-stratified analysis of SBP (Table 4, Figure 2). It is notable that the three significant genes of MAGMA analysis for hypertension were also significant in the SBP. As we found in the single variant EWAS, INO80 was significant in the MAGMA analysis of SBP in females. In the sex-stratified analysis of DBP, there was no significant result in the single SNP and set-wise MAGMA analysis.

### 3.3. Results of Heterogeneity Analysis for Detecting Sex Differences in Genetic Susceptibility to Hypertension

In addition to sex-stratified EWASs, we applied Cochrane’s *Q* and *I^2^* for the detection of sex-specific susceptibility loci for hypertension. The clumping of the results was performed with the two measures. If the *p* values were the same between adjacent loci, the higher *I*^2^ was used as the selection criterion. A physical distance of 250,000 base pairs and *r*^2^ < 0.5 between SNPs were also used for clumping the heterogeneity test. When applying the multiple testing correction, the loci where the results of the sex-stratified EWASs were nominally significant (*p* < 0.05) in at least one or both of the sexes were selected. In the sex-stratified EWAS for hypertension, there were 4307 such variants. In the heterogeneity analysis for hypertension, three loci were significant using Bonferroni’s multiple testing correction (*p* = 0.05/4307 = 1.16 × 10^−5^). Among the results, rs11066015 of ACAD10 had the greatest *I*^2^. This SNP was significant in hypertension and SBP only in the male group. These results indicate that rs11066015 is a credible susceptibility locus for hypertension having different genetic effects on the development of hypertension between males and females. The rs2074356 of HECTD4 had previously been identified as a significant variant in the GWAS of waist circumference, esophageal cancer, blood urea nitrogen, and γ glutamyl-transferase level [34,38,39,40]. Although this locus was not significant in sex-stratified analysis, rs11066280 of HECTD4 showed a significant result in the EWAS of hypertension. The HECTD4 gene was significant in the MAGMA analysis of the male group. These findings suggest that the HECTD4 gene has a sex-specific genetic effect in the development of hypertension.

In the heterogeneity analysis of SBP, a single locus was significant for the selection criteria (Table 5). The number of nominally significant loci in both sexes was 4913, so the adjusted *p* value was 1.02 × 10^−5^ (=0.05/4913). The rs11066280 of HECTD4 showed the highest *I*^2^ score (=94.99) among the results. This locus showed exome-wide significance in the analysis of the male group for hypertension. These results strongly suggest that rs11066280 has an important role in the sex-specific effect on the development of hypertension.

### 3.4. Functional Annotation of Genes Mapped by Nominally Significant Variants in Heterogeneity Analysis

We applied the list of genes that were mapped by nominally significant loci in the Cochrane *Q* test to the FUMA website for the identification of loci that have differential genetic effects between males and females in hypertension, SBP, and DBP. For this purpose, we focused on the enrichment results of the GWAS catalog gene sets and the Kyoto Encyclopedia of Genes and Genomes (KEGG) pathways.

**Figure 3:**
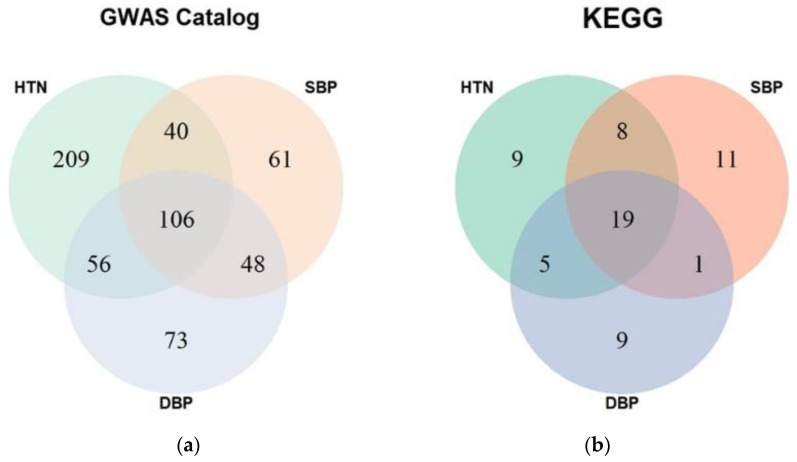
Venn diagrams of results from enrichment analysis with different phenotypes. The numbers indicate common gene sets occurring in results of different phenotypes. (**a**) GWAS catalog gene set, (**b**) KEGG pathway gene set. HTN, hypertension; SBP, systolic blood pressure; DBP, diastolic blood pressure; GWAS, genome-wide association study; KEGG, Kyoto Encyclopedia of Genes and Genomes.

In general, many gene sets are concurrently significant in hypertension, SBP, and DBP results (Figure 3). When the enrichment test was performed with the GWAS catalog gene sets, 106 gene sets were significant in the three groups. The results of enrichment with the KEGG pathways showed better consistency. The ratio between the number of pathways that were significant in the three phenotypes and the number of pathways significant in only one phenotype is greater in the results with KEGG pathways (Figure 3).

**Figure 4:**
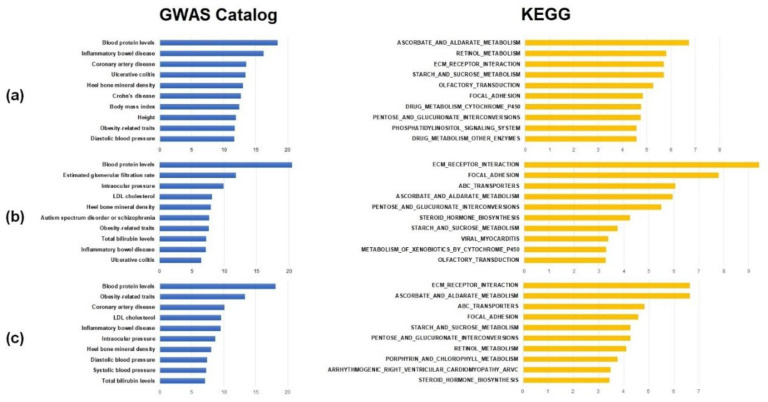
Top 10 results of functional enrichment test with GWAS catalog and KEGG pathway gene sets. The x-axis indicates −log_10_ (*p*-value of enrichment). In the (**a**–**c**), results from hypertension, systolic blood pressure, and diastolic blood pressure are listed, respectively.

Figure 4 shows the top ten significant results of the functional annotation. More detailed results are listed in Supplementary Tables S1 and S2. Among the GWAS catalog gene sets, ‘Blood protein levels’ that are associated with levels of various blood proteins had the most significant results associated with hypertension (adjusted *p* = 3.85 × 10^−19^, Supplementary Table S1). In addition, gene sets that included ‘Height’, ‘Body mass index’, ‘Diastolic blood pressure’, and ‘Obesity-related traits’ that are related to the development of hypertension were found in the top ten significant results. These results indicate that previously identified loci from GWASs of traits that are associated with hypertension might have differential susceptibility between males and females. In the gene set enrichment analysis of KEGG pathways, many pathways that were biologically consistent with known mechanisms of hypertension were significant. Interestingly, ‘Olfactory transduction’ was the most significant pathway in the result (adjusted *p* = 3.85 × 10^−19^, Supplementary Table S2). Because previous studies reported that olfactory receptors of kidney tubules are associated with blood pressure [41], it is possible that the olfactory receptor transduction pathway, which has sex-specific genetic effects, might also play a role in hypertension. The ‘Ascorbate and aldarate metabolism’ pathway, that had the second most significance in the enrichment analysis, was also associated with hypertension. This observation is unsurprising considering previous reports that vitamin C supplementation changes BP [42]. The notable pathway in the results was ‘Steroid hormone biosynthesis’ (Supplementary Table S2, adjusted *p* = 2.76 × 10^−4^). The synthesis of steroid hormones, especially that of sex hormones including estrogen and progesterone, is different between males and females. Although it is not ranked in the top ten significant results, the *p*-value was highly significant. This finding suggests that a sex-specific genetic effect is directly involved in the development of hypertension.

In the gene set enrichment analysis of genes that showed nominal significance in the heterogeneity test of SBP, there were many GWAS catalog phenotype terms and pathways that overlapped with the hypertensive phenotype. In the results, five of the top ten significant enrichment results with GWAS catalog gene sets were significant in both hypertension and SBP. The ‘Blood protein level’ gene set was the most significantly enriched term in the heterogeneity analysis of SBP (adjusted *p* = 3.13 × 10^−21^), as in the results of hypertension. The ‘Low-density lipoprotein (LDL) cholesterol’, ‘Ulcerative colitis’, and ‘Inflammatory bowel disease’ terms were also ranked in the top ten significant pathways in the heterogeneity analysis of SBP. The enrichment test with KEGG pathways showed a similar tendency. In total, six pathways showed significant enrichment in hypertension and SBP (Supplementary Table S2). The ‘Steroid hormone biosynthesis’ pathway was ranked in the top ten significant results (adjusted *p* = 5.64 × 10^−5^).

The nominally significant results from the heterogeneity analysis of DBP had the same tendency as those of hypertension and SBP. In particular, the ‘Blood protein levels’ term of the GWAS catalog had the most significant *p* value as was found with the enrichment analysis of hypertension and SBP (adjusted *p* = 8.10 × 10^–19^). Moreover, the six GWAS catalog gene sets overlapped with the enrichment result in the heterogeneity analysis of hypertension. In addition, the ‘Systolic blood pressure’, and ‘Diastolic blood pressure’ gene sets were ranked within the top ten significant results. The result of the KEGG pathway enrichment analysis included six pathways that were listed in the top ten significant results in the enrichment analysis of the heterogeneity analysis of hypertension. The ‘Steroid hormone biosynthesis’ pathway was also included in the results of hypertension and SBP.

## 4. Discussion

In this analysis, we found genetic loci having differential susceptibility to the development of hypertension by sex. Among the results, the rs11066015 of ACAD10 had a sex-specific effect on the development of hypertension. While the locus was not significant in the sex-combined EWAS, it showed exome-wide significance only in the male group. The heterogeneity of the genetic effect of the loci between males and females was also significant with multiple testing corrections. These data indicate that the rs11066015 is the most plausible locus to be involved in sex-specific genetic susceptibility to hypertension in this analysis. Interestingly, the significant result of EWAS for rs11066015 had been reported only in Asian populations even with other phenotypes, and no variant was found in the same region of the genomes of European and African populations in the GWAS catalog database. Therefore, it is possible that the sex difference of genetic susceptibility of rs11066015 on hypertension might be specific to Asian populations. The rs11066015 is a common variant in the Asian population, while the allele frequency of the variant is very rare in other ethnic groups (Supplementary Figure S1). It seems that the significance of the rs11066015 results from the differences in allele frequencies between ethnic groups. The variant has no significance in large-scaled GWASs from international projects such as the International Consortium for Blood Pressure [43]. The ACAD10 is an enzyme that participates in the β-oxidation of fatty acids in mitochondria [44]. The mitochondrial oxidative stress is associated with hypertension, which is mediated by vascular dysfunction [45]. Therefore, it is possible that the association of ACAD10 with hypertension is partly due to the dysregulation of the mitochondrial oxidation process.

In the previous GWASs, several variants of HECTD4 reported their association with various phenotypes including coffee consumption, alcohol consumption, high-density lipoprotein (HDL), glycemic traits, drinking behavior, SBP, DBP, and hypertension [46,47,48,49,50,51,52]. However, these results were all obtained from sex-combined analyses. In this analysis, rs11066280 of HECTD4 showed male-specific significance. The MAGMA analysis showed the same result, i.e., that HECTD4 was significant in the gene-wise EWAS analysis. Although the rs11066280 was not significant in the heterogeneity analysis of hypertension, it showed exome-wide significance in the heterogeneity analysis of SBP that is known as a clinical predictor for incident hypertension [53]. Moreover, the rs2074356 of HECTD4 had significant sex-specific genetic effects. Furthermore, the locus was associated with hypertension in a previous GWAS [33]. HECTD4 is an E3 ubiquitin ligase that is associated with androgen receptor promotion in prostate cancer cell lines [54]. Although there is no research about the role of HECTD4 in the development of hypertension, the ubiquitin system is known to be involved in the control of blood pressure [55]. These results suggest that HECTD4 is associated with a sex-specific genetic effect on hypertension.

The sex-stratified MAGMA analysis of hypertension and SBP indicate that ALDH2 is another possible variant having a significant effect on hypertension in the male population. SNPs in ALDH2 are significantly associated with hypertension. The finding was not replicated in the non-alcohol drinking population, and alcohol consumption is known to affect the genetic effect of the ALDH2 variants. Moreover, it has already been reported that the GG genotype of rs671 of ALDH2 is associated with an increased risk of hypertension, especially in men. Therefore, ALDH2 is highly likely to have a male-specific genetic effect on the development of hypertension, even though the SNP was not significant in the heterogeneity test. The ALDH2 gene has a functional implication in the development of hypertension. The biological role of ALDH2 transforms acetaldehyde into acetic acid, and it has a protective function against oxidative stress [56]. Since the excessive generation of reactive oxygen species (ROS) seems to have a critical role in the pathogenesis of hypertension [45], the genetic variation of ALDH2 might be associated with the dysregulation of the ROS, which results in hypertension.

In the heterogeneity test, the variants of ACAD10 and HECTD4 showed opposite directions of genetic effects between male and female. Since the genes are involved in the oxidation and ubiquitination process, it is possible that sex hormones such as estrogen are responsible for the effects considering the results of previous studies. For example, estrogen can provide nitric oxide or superoxide products according to the conditions of vascular walls [57]. The main function of the epithelial sodium channel (ENaC) in the kidney distal nephron, which mediates sodium and water balance and the stabilization of blood pressure, is affected by estrogen effects of increasing ubiquitination [58]. These seem to support the results of the heterogeneity test.

In the sex-stratified EWAS, the rs142469845 of INO80 is significant in the female group. The variant is a rare variant, and its allele frequency is very low in the other populations (Supplementary Figure S2). Moreover, the rs112925537 and rs28866311 of INO80 are significantly associated with blood pressure in other GWASs (Supplementary Table S3), and a rare mutation (Ser818Cys) of INO80 was found in a syndrome of abnormal aortic aging and systolic hypertension [59]. Although further validation is required, these indicate that the rs142469845 is a genuine rare variant having susceptibility to hypertension in the female population.

In the functional enrichment analysis, we used nominal results (*p* < 0.05) of the heterogeneity analysis to identify the biological implications of sex differences in genetic susceptibility to hypertension and BP. Because false-positive results might be included in the nominal results, it is expected that such analysis can indicate an overall tendency in biological functions of genetic variants having sex differences in susceptibility to hypertension. In the results, pathways that are related to hypertension, SBP, and DBP were consistently found in GWAS catalog gene sets and KEGG pathways, especially in the top ten significant results. Of the results, those gene sets such as ‘Body mass index’, and ‘Obesity-related traits’ are predisposing conditions to hypertension [60]. It was interesting that the ‘Hypertension’ gene set was not highly significant in the enrichment analysis with the results of the heterogeneity analysis in hypertension (adjusted *p* = 0.03, Supplementary Table S1). This might indicate that previously identified genetic loci for hypertension are composed of a mixture of variants that have a genetic effect that is common to both sexes as well as differential genetic effects on the development of hypertension. In the top ten results of the GWAS catalog gene set enrichment, ‘Inflammatory bowel disease (IBD)’ gene sets including ‘Inflammatory bowel disease’, ‘Crohn’s disease’, and ‘Ulcerative colitis’ gene sets overlapped in the results of different phenotypes. Because hypertension tends to occur more in IBD patients [61], it is possible that tentative protective effects of IBD-related variants on hypertension are dependent on sex differences.

In the significant results from the enrichment test with KEGG pathways, the ‘Steroid hormone receptor biosynthesis’ pathway seems to strongly indicate that genetic variants affect the development of hypertension differentiated by sex. The pathway was significant in the heterogeneity test of all different phenotypes (hypertension, SBP, and DBP), and the heterogeneity was measured in terms of susceptibility to hypertension. Therefore, variants having sex-specific genetic susceptibility may be related to genes involved in the pathway. In previous research, steroid hormones were shown to be related to hypertension [62]. Thus, it seems that the effect of steroid hormone biosynthesis on hypertension might differ according to sex. Given these observations, there is a possibility that previously known genetic loci for hypertension and BP might also express sex differences in their effects on phenotype.

## 5. Conclusions

In this study, we identified loci genetically susceptible to hypertension that have different effects on hypertension between males and females using exome array data from a Korean population. Even with a relatively marginal number of samples, we could identify significant exome-wide loci for hypertension and BP. In addition, a sex-stratified EWAS of approximately half of all samples found genetic susceptibility loci having a sex-specific effect on hypertension or BP. Some of these loci also showed significance in the heterogeneity analysis. The gene set enrichment analysis of nominally significant loci in the heterogeneity analysis of hypertension and BP showed substantial consistency between results. These data indicate that there is a high possibility of differences in genetic effects on hypertension based on sex. Their characterization is essential for understanding the sex-specific pathophysiology of hypertension.

## Figures and Tables

**Table 1:** Exome-wide association study results of hypertension in total, male, and female population.

	Chr	BP	SNP	Gene	MA	MAF	OR (95% CI)	*p*-Value
Total population (*n* = 13,926)	4	81184341	rs16998073	PRDM8, FGF5	A	0.34	1.22 (1.16–1.30)	4.46 × 10^−12^
	10	104719096	rs12413409	CNNM2	A	0.24	0.82 (0.77–0.88)	2.79 × 10^−9^
	12	90008959	rs2681472	ATP2B1	G	0.38	0.86 (0.82–0.91)	3.59 × 10^−7^
Male group (*n* = 6402)	12	112168009	rs11066015	ACAD10	A	0.16	0.73 (0.65–0.81)	7.99 × 10^−9^
	12	112817783	rs11066280	HECTD4	A	0.17	0.75 (0.68–0.84)	1.26 × 10^−7^
	4	155804083	rs1392550	RBM46, NPY2R	G	0.40	1.22 (1.13–1.32)	8.77 × 10^−7^
Female group (*n* = 7524)	4	81184341	rs16998073	PRDM8, FGF5	A	0.34	1.25 (1.15–1.35)	1.18 × 10^−7^

*n*, number of samples; Chr, chromosome; BP, base-pair position; SNP, single-nucleotide polymorphism; MA, minor allele; MAF, minor allele frequency; OR, odds ratio; CI, confidence interval.

**Table 2:** Results of hypertension MAGMA analysis in the total and male population.

	Gene Symbol	Chr	Start	Stop	nSNP	Stat	*p*-Value
Total population	CNNM2	10	104677050	104850978	39	5.5413	1.50 × 10^−8^
	CYP17A1	10	104589288	104598290	34	5.4642	2.33 × 10^−8^
	AS3MT	10	104628273	104662656	37	5.4113	3.13 × 10^−8^
	C10orf32-ASMT	10	104613029	104662656	36	4.5921	2.19 × 10^−6^
Male group	ALDH2	12	112204691	112247782	32	5.6245	9.30 × 10^−9^
	HECTD4	12	112597992	112819896	34	5.4362	2.72 × 10^−8^
	ACAD10	12	112123857	112194903	33	4.4441	4.41 × 10^−6^

Chr: chromosome; Start, start position of a gene; Stop, end position of a gene; nSNP, number of SNPs; Stat, statistics of *p*-value summation in the MAGAM analysis.

**Table 3:** Sex-stratified EWAS results of systolic blood pressure.

	Chr	BP	SNP	Gene	MA	MAF	β (95% CI)	*p*-Value
Male group (*n* = 5409)	12	112168009	rs11066015	ACAD10	A	0.16	−2.02 (−2.76 to −1.29)	7.34 × 10^−^^8^
	12	112817783	rs11066280	HECTD4	A	0.17	−1.94 (−2.65 to −1.22)	1.05 × 10^−7^
Female group (*n* = 6255)	15	41276477	rs142469845	INO80	A	1.42 × 10^−4^	42.93 (26.13–59.74)	1.18 × 10^−7^

*n*, number of samples; Chr, chromosome; BP, base-pair position; SNP, single-nucleotide polymorphism; MA, minor allele; MAF, minor allele frequency; OR, odds ratio; CI, confidence interval.

**Table 4:** Results of sex-stratified MAGMA analysis for systolic blood pressure.

	Symbol	Chr	Start	Stop	nSNP	Stat	*p*-Value
Male group	ALDH2	12	112204691	112247782	32	5.1947	1.03 × 10^−7^
	HECTD4	12	112597992	112819896	34	5.0576	2.12 × 10^−7^
	ACAD10	12	112123857	112194903	33	4.8091	7.58 × 10^−7^
Female group	INO80	15	41271078	41408552	26	4.8509	6.15 × 10^−7^

Chr: chromosome; Start, start position of a gene; Stop, end position of a gene; nSNP, number of SNPs; Stat, statistics of *p* value summation in the MAGAM analysis.

**Table 5:** Significant results in the heterogeneity test of hypertension and systolic blood pressure.

	Chr:BP	SNP	Gene	OR_M (95% CI)	OR_F (95% CI)	*I* ^2^	*p*-Value
HTN	12:112168009	rs11066015	ACAD10	0.73 (0.65–0.81)	1.09 (0.99–1.22)	96.49	2.22 × 10^−6^
	12:112645401	rs2074356	HECTD4	0.74 (0.66–0.83)	1.09 (0.98–1.22)	95.77	1.11 × 10^−5^
SBP	12:112817783	rs11066280	HECTD4	−1.94 (−2.65–1.22)	0.35 (−0.36–1.05)	94.99	7.93 × 10^−6^

HTN, hypertension; SBP, systolic blood pressure; Chr, chromosome; BP, base position; SNP, single-nucleotide polymorphism; OR_M, odds ratio in male population; OR_F, odds ratio in female population; *I*^2^, heterogeneity index.

## Data Availability

The exome array data is available from the National Biobank of Korea.

## Supplementary Materials

The following are available online at https://www.mdpi.com/article/10.3390/genes12111804/s1, Figure S1: Search result of rs11066015 in dbSNP, Figure S2: Search result of rs142469845 in dbSNP, Table S1: Results of functional enrichment tests with GWAS catalog gene sets, Table S2: Results of functional enrichment tests with KEGG pathway gene sets, Table S3: Variants of INO80 in GWAS catalog database.
