# Supplementary material for: A Genome-Wide Association Study of a Korean Population Identifies Genetic Susceptibility to Hypertension Based on Sex-Specific Differences
Source: Genes (Basel). 2021 Nov 16;12(11):1804. doi: 10.3390/genes12111804 (PMC8622776; doi:10.3390/genes12111804)
Supplement: Supplementary file 1 [file genes-12-01804-s001.zip › genes-1446663-Supplementary.pdf]

rs11066015

Current Build 155
Released April 9, 2021

Organism *Homo sapiens*

Position chr12:111730205 (GRCh38.p13)

Alleles G>A

Variation Type SNV Single Nucleotide Variation

Frequency A=0.008954 (2370/264690, TOPMED)
A=0.009846 (1781/180884, ALFA)
A=0.005964 (836/140180, GnomAD) (+ 14 more)

Clinical Significance Not Reported in ClinVar

Gene : Consequence ACAD10 : Intron Variant

Publications 7 citations
LitVar 16

Genomic View See rs on genome

Population	Group	Sample Size	Ref Allele	Alt Allele
Total	Global	180884	G=0.990154	A=0.009846
European	Sub	158164	G=0.999848	A=0.000152
African	Sub	4578	G=0.9993	A=0.0007
African Others	Sub	180	G=1.000	A=0.000
African American	Sub	4398	G=0.9993	A=0.0007
Asian	Sub	6340	G=0.8058	A=0.1942
East Asian	Sub	4492	G=0.7870	A=0.2130
Other Asian	Sub	1848	G=0.8517	A=0.1483
Latin American 1	Sub	504	G=1.000	A=0.000
Latin American 2	Sub	1092	G=0.9991	A=0.0009
South Asian	Sub	286	G=1.000	A=0.000

Supplementary Figure S1. Search result of rs11066015 in dbSNP. Note that minor allele frequency is high in Asian population, while it is very low in the other ethnic groups.

rs142469845

Current Build 155
Released April 9, 2021

Organism *Homo sapiens*
Position chr15:40984279 (GRCh38.p13)
Alleles G>A
Variation Type SNV Single Nucleotide Variation
Frequency A=0.000442 (117/264690, TOPMED)
A=0.000338 (85/251310, GnomAD_exome)
A=0.000148 (25/168560, ALFA) (+ 7 more)

Clinical Significance Not Reported in ClinVar
Gene : Consequence INO80 : Missense Variant
Publications 0 citations
Genomic View See rs on genome

Frequency | Variant Details | Clinical Significance | HGVS | Submissions | History | Publications | Flanks

ALFA Allele Frequency

The ALFA project provide aggregate allele frequency from dbGaP. More information is available on the project page including descriptions, data access, and terms of use.

Release Version: 20201027095038

Search:

Population	Group	Sample Size	Ref Allele	Alt Allele
**Total**	Global	168560	G=0.999852	A=0.000148
European	Sub	146864	G=1.000000	A=0.000000
African	Sub	4292	G=0.9986	A=0.0014
African Others	Sub	168	G=1.000	A=0.000
African American	Sub	4124	G=0.9985	A=0.0015
Asian	Sub	6294	G=0.9981	A=0.0019
East Asian	Sub	4476	G=0.9987	A=0.0013

Supplementary Figure S2. Search result of rs142469845 in dbSNP. The minor allele frequency of the variant is very low in all ethnic groups.
